# A comprehensive analysis of non-sequential alignments between all protein structures

**DOI:** 10.1186/1472-6807-7-78

**Published:** 2007-11-16

**Authors:** Alexej Abyzov, Valentin A Ilyin

**Affiliations:** 1 Department of Biology, Northeastern University 360 Huntington Avenue, Boston, MA 02115, USA

## Abstract

**Background:**

The majority of relations between proteins can be represented as a conventional sequential alignment. Nevertheless, unusual non-sequential alignments with different connectivity of the aligned fragments in compared proteins have been reported by many researchers. It is interesting to understand those non-sequential alignments; are they unique, sporadic cases or they occur frequently; do they belong to a few specific folds or spread among many different folds, as a common feature of protein structure. We present here a comprehensive large-scale study of non-sequential alignments between available protein structures in Protein Data Bank.

**Results:**

The study has been conducted on a non-redundant set of 8,865 protein structures aligned with the aid of the TOPOFIT method. It has been estimated that between 17.4% and 35.2% of all alignments are non-sequential depending on variations in the parameters. Analysis of the data revealed that non-sequential relations between proteins do occur systematically and in large quantities. Various sizes and numbers of non-sequential fragments have been observed with all possible complexities of fragment rearrangements found for alignments consisting of up to 12 fragments. It has been found that non-sequential alignments are not limited to proteins of any particular fold and are present in more than two hundred of them. Moreover, many of them are found between proteins with different fold assignments. It has been shown that protein structure symmetry does not explain non-sequential alignments. Therefore, compelling evidences have been provided that non-sequential alignments between proteins are systematic and widespread across the protein universe.

**Conclusion:**

The phenomenon of the widespread occurrence of non-sequential alignments between proteins might represent a missing rule of protein structure organization. More detailed study of this phenomenon will enhance our understanding of protein stability, folding, and evolution.

## Background

Protein structure comparison is a fundamental approach in many areas of biomedical studies. Its applications range from protein classification and establishing evolutionary relationship between proteins to functional prediction, molecular modeling and protein engineering. While structure comparison can be done in a number of ways, protein structure alignment is one of the major techniques used, populated today with more than 40 methods, the most complete list of which can be found at Wikipedia [1]. These methods rely on a wide variety of statistical, geometrical, physical, and other structure properties in order to produce an alignment. But most of them follow a simple sequential rule: two proteins are aligned in sequential order, by placing their chains adjacent to each other from N-terminal to C-terminal and introducing gaps.

**Figure 1:**
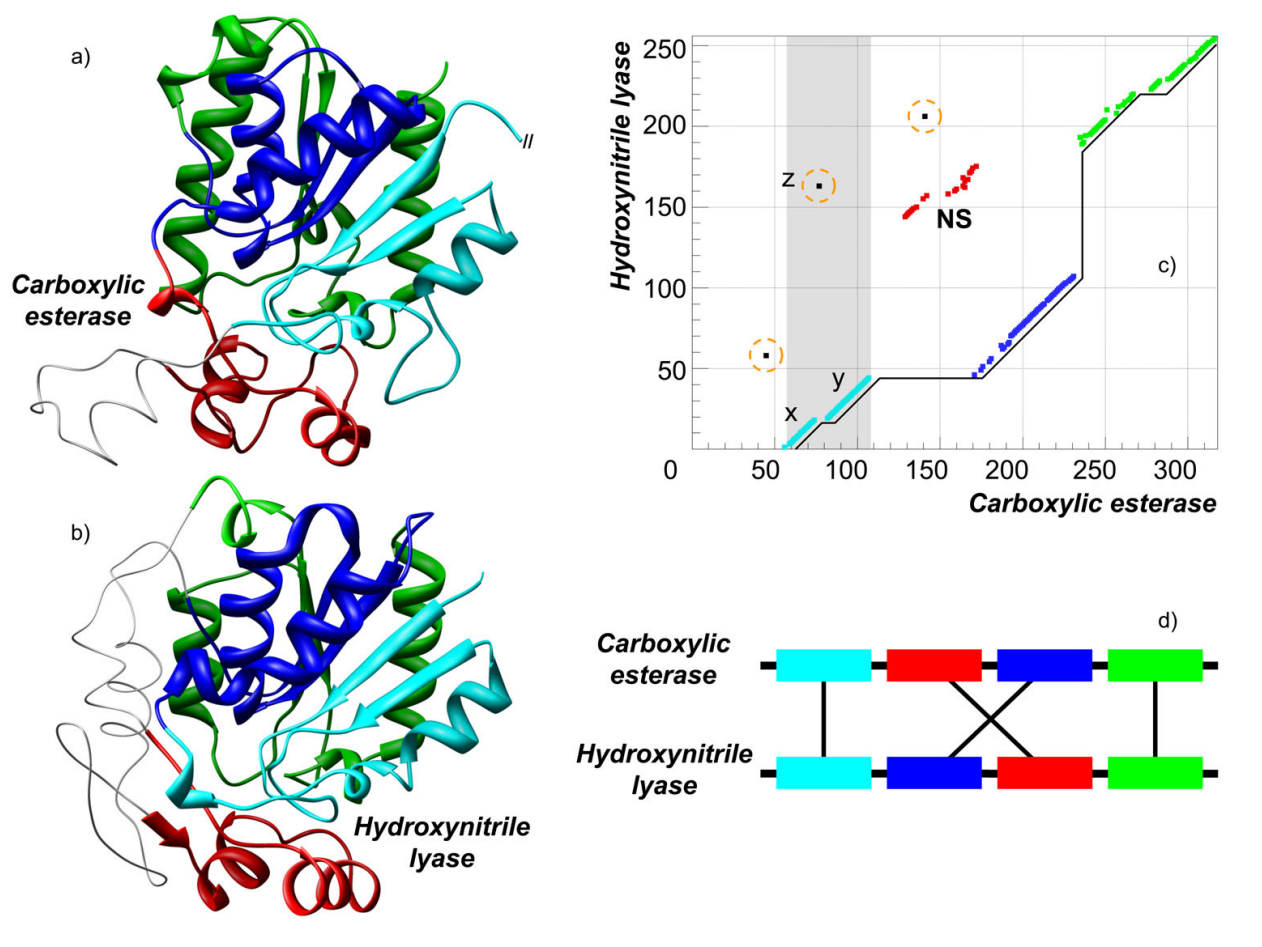
**Example of a non-sequential alignment**. Structures of hydroxynitrile lyase from *Hevea brasiliensis *(PDB-code 1yas:A, shown on a) and esterase from *Alcaligenes sp*. (PDB-code 1qlw:A, shown on b) have been aligned by TOPOFIT with *N*_*e*_/*RMSD *of 175/1.7 Å. The corresponding alignment plot is shown on c), the connectivity of the aligned fragments is shown on d). The structural alignment consists of 7 fragments, which can be combined into 4 segments colored in cyan, blue, red and green. Three obvious cases of noise in the alignment are circled. A continuous line represents the longest sequential alignment and the non-sequential segment is termed NS. The figure also shows how the noise fragment termed ***z ***interferes with the two long fragments, ***x ***and ***y ***(grayed area).

The key representation of such sequential alignment was introduced as a matrix approach by Needleman and Wunsch [2], which states that given a scoring function, the optimal alignment is the best way through the matrix. Such an approach has fertilized a large number of methods on sequence and structure alignments and resulted in many achievements in our understanding of protein similarities, their evolutionary relationships, functionality and so on. However, there is a number of cases reported in literature, which are unusual from the sequential point of view, for which structurally equivalent parts have different connectivity in the sequences of compared proteins. These alignments cannot be represented as a diagonal path through the matrix. Figure 1 shows an example of such an alignment. The alignment consists of four segments; only three of them can be included in a sequential alignment. Since the remaining segment is a part of the alignment, but is not in a sequential order, it is called non-sequential (NS); accordingly, the alignment is called non-sequential. A non-sequential alignment is an alignment where structurally similar parts are not in the same order in protein sequences.

Understanding more about these types of alignments is interesting; are they unique, sporadic cases; do they occur frequently; do they belong to a few specific folds or spread among many different folds as a common feature of protein structure. Such a large-scale study is also important for the theoretical understanding of protein organization, the evolution of proteins, and using non-sequential approach has a practical application as a designing tool in protein engineering.

Many researches have reported cases of non-sequential alignments such as circular permutations, domain or region swaps [3-15], and *β*-hairpin flip [6,10]. The most studied case of non-sequential alignments is a circular permutation, when the N-terminal of each aligned protein is aligned with the C-terminal of the other protein. The circular permutations have been analyzed by both sequence and structure related computational methods [16,17]. A suggested evolutionary mechanism for circular permutation in proteins [18] states that first a gene duplication of the precursor gene occurs in such a way that both genes become fused in frame, leading to a tandem protein. After generation(s) of a new start codon within the 5' part of the tandem gene and a stop codon at an equivalent position in the 3' part of the gene, a protein is encoded that represents a circular permutation of the precursor gene product. Later the mechanism was shown to be valid for a protein family of adenine-n6 DNA methyltransferases [19]. Many naturally occurring proteins were experimentally redesigned to have circular permutation and it was shown that they preserve their structure and function [20-30]; thus providing evidence that circular reordering of protein structural elements does not affect protein folding and functionality.

The appearance of similar domains/regions in different orders in sequence as a domain/region swap have been analyzed by Fliess and coworkers [31]. Their study was based on sequence alignments of proteins in the Swiss-Prot database [32], where they found 140 swap cases and concluded that the swapping of regions is a relatively rare evolutionary event. A comparatively large (at that time) structure based large-scale analysis of non-sequential cases has been reported about a decade ago [4], where 426 representative structures from PDB were analyzed by the SARF2 method. Along with other results, that work presented several cases of non-sequential alignments and estimated that they are found in 11% of cases.

Since then several methods for protein structure alignment have been developed which can produce non-sequential alignments [15,33-38] including TOPOFIT [39], developed in our group. MASS [34] method was developed to produce multiple structure alignments; GANGSTA [36] and SCALI [15] were suggested to be used for structure classification; SSM [35] and KENOBI [33] appear to be computationally efficient and OPAAS [37] was applied to analysis of alternative structure alignments. TOPOFIT compares topologies of Delaunay tessellation patterns calculated using positions of C*α*-atoms in protein structures and does not assume any sequential order of residues in an alignment. Its distinctive feature is that the method does not balance between lower *RMSD *and a higher number of aligned positions (*N*_*e*_) but rather identifies the largest group of residues which have the same neighbors in the same locations common in both compared structures, defined mathematically as a topological invariant and detected by saturation point (topomax point) in the spatial tessellation graph. Such an objective methodology provides unambiguous identification and separation of the structurally invariant parts from the variable parts by identifying a precise border between the two. Unlike all other methods (which can produce non-sequential alignments), composing alignments of fragments or secondary structure elements, TOPOFIT extends an alignment pair by pair of residues; thus, is not biased by fragment choice or secondary structure element definition. The method is also computationally efficient, so that all proteins in the PDB (as of July 2005) have already been calculated, grouped into clusters and stored in the TOPOFIT-DB database [40]. We have used TOPOFIT in our comprehensive large-scale analysis of non-sequential relations between proteins. To the best of our knowledge this is the first comprehensive large-scale analysis of non-sequential alignments between all available protein structures.

## Results

### Non-sequential alignments between proteins do occur systematically and in large quantities

**Figure 2:**
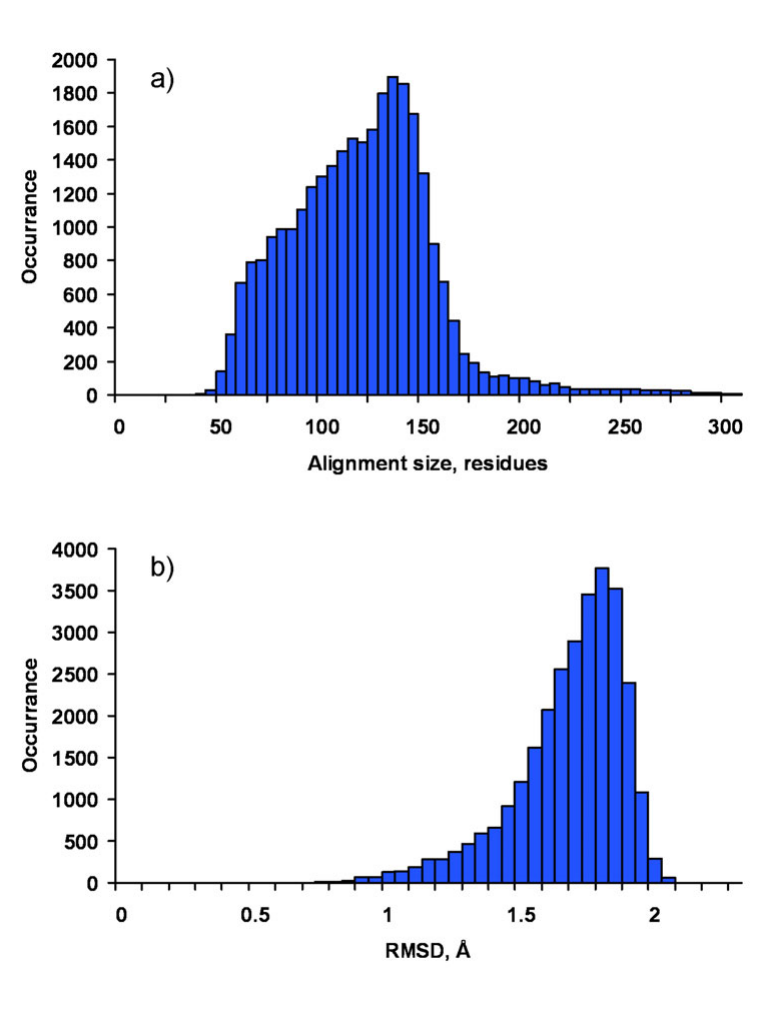
Distribution of alignment size (shown on a) and RMSD (shown on b) of the 28,949 non-sequential alignments analyzed in the D1 dataset.

A comprehensive large-scale analysis of 8,865 non-redundant representatives from each protein cluster in TOPOFIT-DB [40] has been performed. TOPOFIT-DB is a collection of alignments for all significant values of *Z-score*, i.e. *Z-score *> 3. From the experience of using T-DB we should mention that the range of *Z-score *values from 3 to 5 is the "twilight zone" where together with structurally significant alignments there are also trivial cases containing just one or two secondary structure elements; while alignments at *Z-score *> 5 typically represent high structural similarity between proteins. But to ensure the validity of this study we used an even tighter criteria: only the alignments with very high structural similarity, *Z-score *> 7, have been collected, resulting in total of 82,263 structurally similar protein pairs. These alignments are referred to as dataset D1. The alignments collected in the dataset D1 are considerably large in size (with average of 120 aligned amino acid residues) and represent high structural match (*RMSD *< 2 Å) as shown in Figure 2. Thus, there is no doubt of their structural similarity.

Another dataset has been collected by compiling alignments between protein families as defined by SCOP [41] (release 1.69). For each family, the first structure in the list of proteins for the corresponding family has been used as a representative, resulting in 2,845 representatives. 4,045,590 structural alignments have been produce and stored in TOPOFIT_DB database [40] by comparing the representatives. As for dataset D1 only alignments with *Z-score > 7 *have been used, resulting in total of 4,648 alignments. The distributions of their alignment sizes and *RMSD *are similar to the ones for dataset D1. These alignments will be referred to below as dataset D2.

The most striking and surprising result from the analysis performed here is that **non-sequential (NS) alignments have been found in large quantities in structurally similar proteins**. In other words, there are many alignments between highly structurally similar proteins for which the alignment matrix is not diagonal. The overall proportion of non-sequential alignments was estimated to be as high as 35.2%, but not lower than 17.4% when tightened thresholds have been applied (see details later in Table 1). The detected non-sequential alignments are presented in a large variety of alignment patterns with various orders of alignment fragments in structurally similar proteins, as well as with various sizes and numbers of non-sequential fragments. They can be as simple as an almost sequential alignment with the rearrangement of a single fragment, and as complex as it is hard to define what the sequential part in the alignment is. Even more interesting, many cases of reverse alignments have been detected, i.e. alignments where fragments structurally match each other but the polypeptide chains go in opposite directions.

**Table 1:** General statistics on non-sequential cases.

Best alignment: #28,949 (35.2%) (dataset D1)				Alternative: 17,428 (21.2%) (dataset D1)				Alternative with tightened (dataset D1)	Alternative with tightened (dataset D2)
F	18,701 (22.7%)	C	15,933(19.4%)	9,226 (11.2%)	C	11,742 (14.3%)	F	10,818 (13.2%)	717(15.4%)
		W	1,949 (2.4%)	2,008 (2.4%)	W				
		X	819 (1.0%)	508 (0.6%)	X				

		S	2,324 (2.8%)	1,901 (2.3%)	S	5,438 (6.6%)	M	3,164 (3.9%)	192 (4.1%)
M	8,764 (10.7%)	C	867 (1.1%)	315 (0.4%)	C				
		W	1,925 (2.3%)	1,030 (1.3%)	W				
		X	3,648 (4.4%)	2,192 (2.7%)	X				

R	1,484 (1.8%)	S	497 (0.6%)	139 (0.2%)	S	248 (0.3%)	R	252(0.3%)	26 (0.6%)
		C	695 (0.8%)	59 (0.1%)	C				
		W	224 (0.3%)	42 (0.1%)	W				
		X	68 (0.1%)	8 (0.0%)	X				

Annotation of classes: F is forward, M is mixed and R is reverse. Annotation of subclasses: S is simple, C is circular, W is swap and X is complex. The column "Best alignment" shows numbers obtained using the best (largest) alignment, while the column "Alternative" shows the statistics calculated when trivial cases of non-sequential alignment have been eliminated using alternative alignments, as described in the text. The last two columns show the statistics when tightened criteria have been used. There is no line for forward simple alignments as they are sequential.

### Types of observed non-sequential alignments

**Figure 3:**
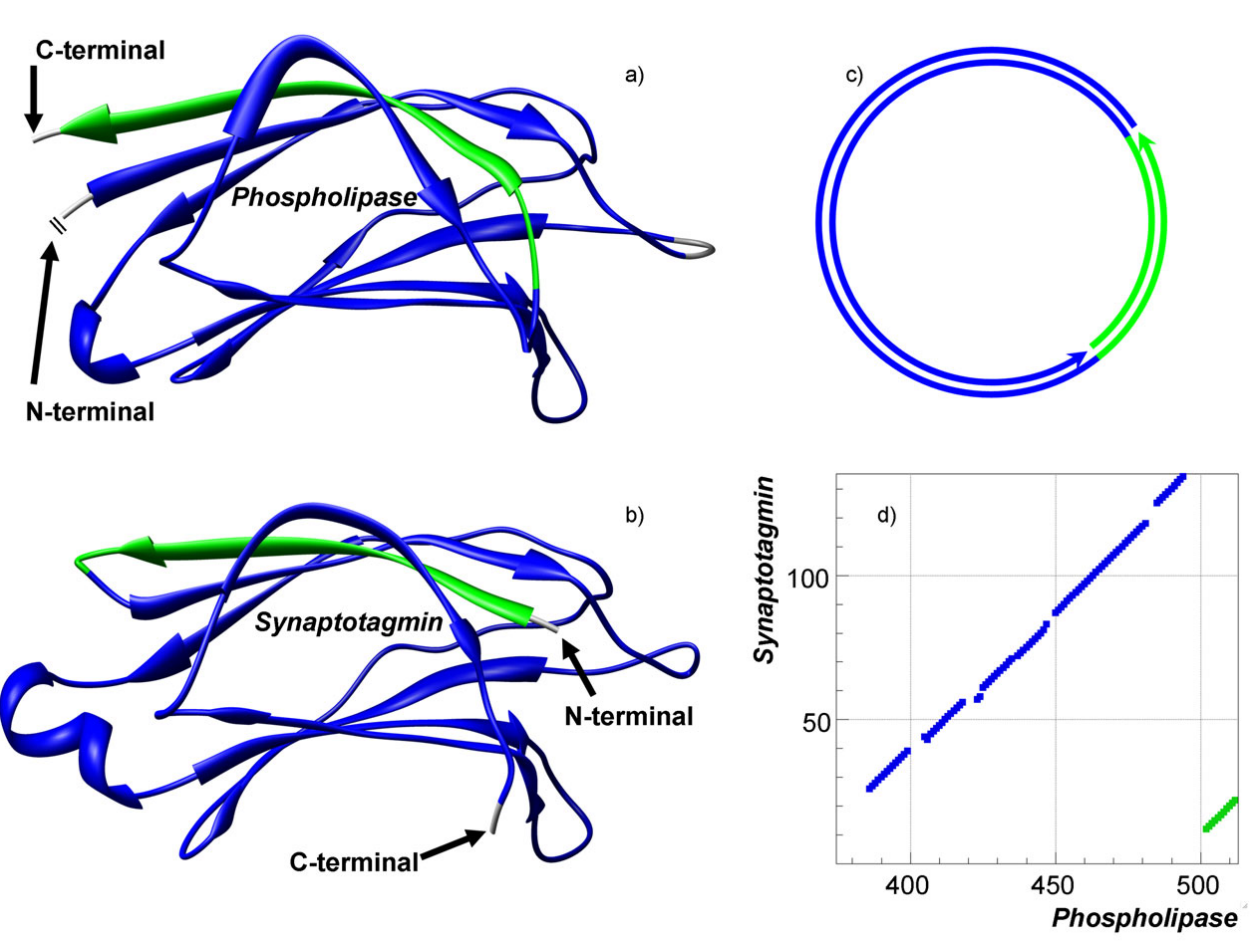
**Example of a circular permutation in a non-sequential alignment**. Structures of posphoinositide-specific phospholipase C delta (PDB-code 2isd:A, shown on a) and C2-domain of synaptotagmin I (PDB-code 1rsy, shown on b) have been aligned by TOPOFIT with the *N*_*e*_*/RMSD *of 108/1.2 Å. The alignment consists of two segments colored in blue and green. The green segment represents a *β*-strand and is located at N-terminal in synaptotagmin and at C-terminal in phospholipase. Thus the alignment is the circular permutation. c) displays the circular diagram of the alignment. d) displays the alignment plot corresponding to the alignment.

The easiest and also the most studied case of non-sequential alignment is a circular permutation, which is defined as a case where the structurally equivalent part of a protein has been rearranged from N- to C-terminal (or vise versa) in the protein sequence. An example of a circular permutation alignment for posphoinositide-specific phospholipase C delta (PDB-code 2isd:A) and C2-domain of synaptotagmin I (PDB-code 1rsy) is shown in Figure 3 (both proteins are from *Rattus norvegicus*). The structures are aligned at *N*_*e *_= 108 and *RMSD *= 1.2 Å, where *N*_*e *_is number of equivalent residues in alignment and *RMSD *is root mean square deviation between C*α*-atoms of the equivalent residues; and the alignment consist of two parallel layers of 4 *β*-strands. In synaptotagmin one of the *β*-strands is located at the N-terminal end, while in phospholipase, its structural equivalent is at the C-terminal end. This *β*-strand is the non-sequential part of the alignment and can be seen on the alignment plot as a small fragment (in green) parallel to the long sequential alignment (Figure 3d).

**Figure 4:**
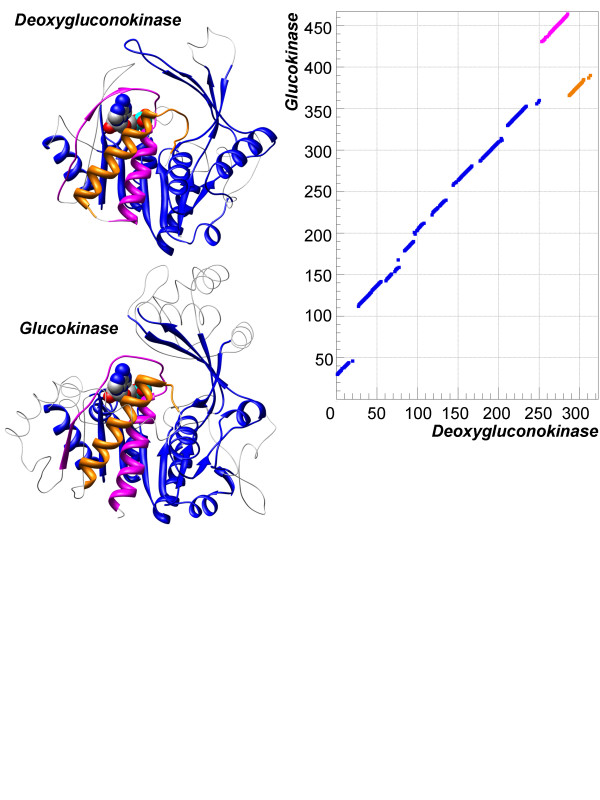
**Example of segment swap in non-sequential alignment**. Structures of glucokinase (PDB-code 1gc5:A) and 2-dehydro-3- deoxygluconokinase (PDB-code 1j5v:D) have been aligned by TOPOFIT with *N*_*e*_*/RMSD *of 234/1.7 Å. Alignment segments are colored in blue, magenta and orange. The right side of the picture displays the corresponding alignment plot. It is easy to see that if the orange and magenta segments would be swapped in either sequence of the compared proteins the result would be a perfect sequential alignment.

Similar to the circular permutations there are also alignments with just one structurally equivalent part rearranged in the sequence, but not necessarily from N- to C-terminal. An example has already been shown in Figure 1, where there is a long sequential alignment, while the non-sequential part (NS) is located in the middle of the alignment. Another example of an alignment of such type is shown in Figure 4, where the structure of 2-dehydro-3-deoxygluconokinase from *Thermus thermophilus *(PDB-code 1v1b) and ADP-dependent glucokinase from *Thermococcus litoralis *(PDB-code 1gc5:A) are aligned at *N*_*e *_= 234 residues and *RMSD *of 1.7 Å. In this example, two structurally equivalent regions: 1) *α*-helix and 2) *α*-helix and *β*-strand are located one after another but in a different order in the sequences of the compared proteins. Most of the alignment is sequential, namely, one can produce a long sequential alignment out of the aligned residues with only a small part of it being non-sequential, either magenta or orange on the picture. It is evident that if those parts were swapped in any of the sequences then one would get a perfect sequential alignment. Based on this observation, we will call such alignments "swaps". Interestingly, the functionality of these proteins is similar and involves ATP/ADP binding. Moreover, the binding site residues are composed from the parts, which are non-sequential.

**Figure 5:**
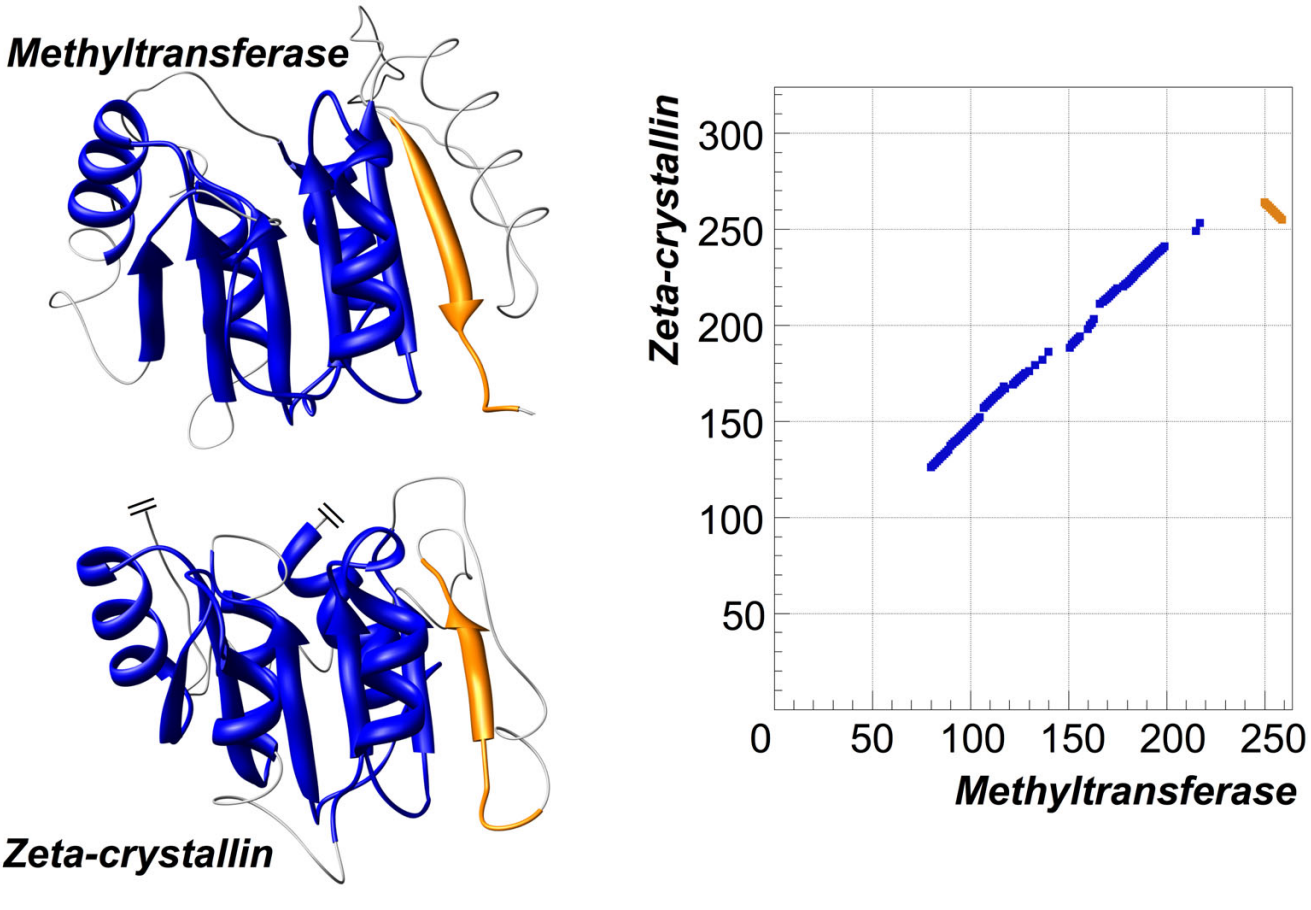
**Example of non-sequential alignment with reverse segment**. Structures of adoment dependent methyltransferase (PDB-code 1i9g:A) and zeta-crystallin (PDB-code 1yb5:A) have been aligned by TOPOFIT with *N*_*e*_*/RMSD *of 115/1.7 Å. The longest sequential alignment is colored in blue. The fragment aligned in reverse order is colored in orange. The right side of the picture displays the corresponding alignment plot.

Another type of simple non-sequential alignment is similar to the above examples, but different in the direction of the polypeptide chain. Such alignment is observed when all the structurally aligned fragments have the same order in the sequences, but the direction of the chains in one fragment is opposite, i.e. in one protein the residues in this fragment go from N- to C-terminal, while in the other protein they go from C- to N-terminal. An example of such alignment is shown in Figure 5 for adoment-dependent methyltransferase from *Mycobacterium tuberculosis *(PDB-code 1i9g:A) and zeta-crystallin from *Homo sapiens *(PDB-code 1yb5:A). These two structures are very similar (RMSD is 1.7 Å) with the non-sequential region found at the place where antiparallel *β*-strand of methyltransferase is aligned to the parallel *β*-strand of zeta-crystallin. There is no permutation of fragment order in these proteins; most of the alignment is sequential while the reverse part, just 10 residues, is small but noticeable. To separate such cases (with opposite direction in the aligned chains) from the previous alignments we will call the aligned fragments with the same direction of the polypeptide chain as the 'forward' alignment and those with the opposite direction as the 'reverse'.

**Figure 6:**
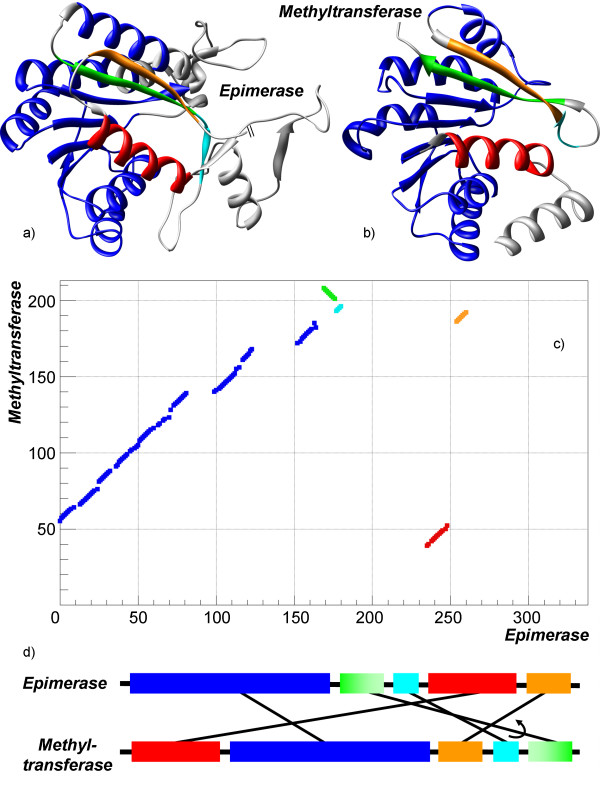
**Example of complex non-sequential alignment**. Structures UDP-galactose 4-epimerase (PDB-code 1kvu, shown on a) andcatechol o-methylstransferase (PDB-code 1vid, shown on b) have been aligned by TOPOFIT with *N*_*e*_*/RMSD *137/1.7 Å. Five alignment segments are shown by different colors; four of them are non-sequential. c) displays an alignment plot corresponding to the alignment. d) displays a schematic linear diagram of segment permutation in the alignment.

More complex examples consist of alignments with several non-sequential fragments, which can be forward and/or reverse. As shown in Figure 6, an alignment of UDP-galactose 4-epimerase from *Escherichia coli *(PDB-code 1kvu) andcatechol o-methylstransferase from *Rattus norvegicus *(PDB-code 1vid) has four non-sequential fragments, one of which is reverse. The two proteins share a large common structural part, consisting of 137 residues superimposed at RMSD of 1.7 Å. The major part of it is the long sequential alignment, while the non-sequential fragments are three secondary structural elements (*α*-helix and two *β*-strands) and an irregular fragment of four residues. Even though the number of residues in the non-sequential fragments (24 residues) is not that large, the permutation of fragments in the sequences of protein is complex, which is shown on the schematic diagram (Figure 6d).

**Figure 7:**
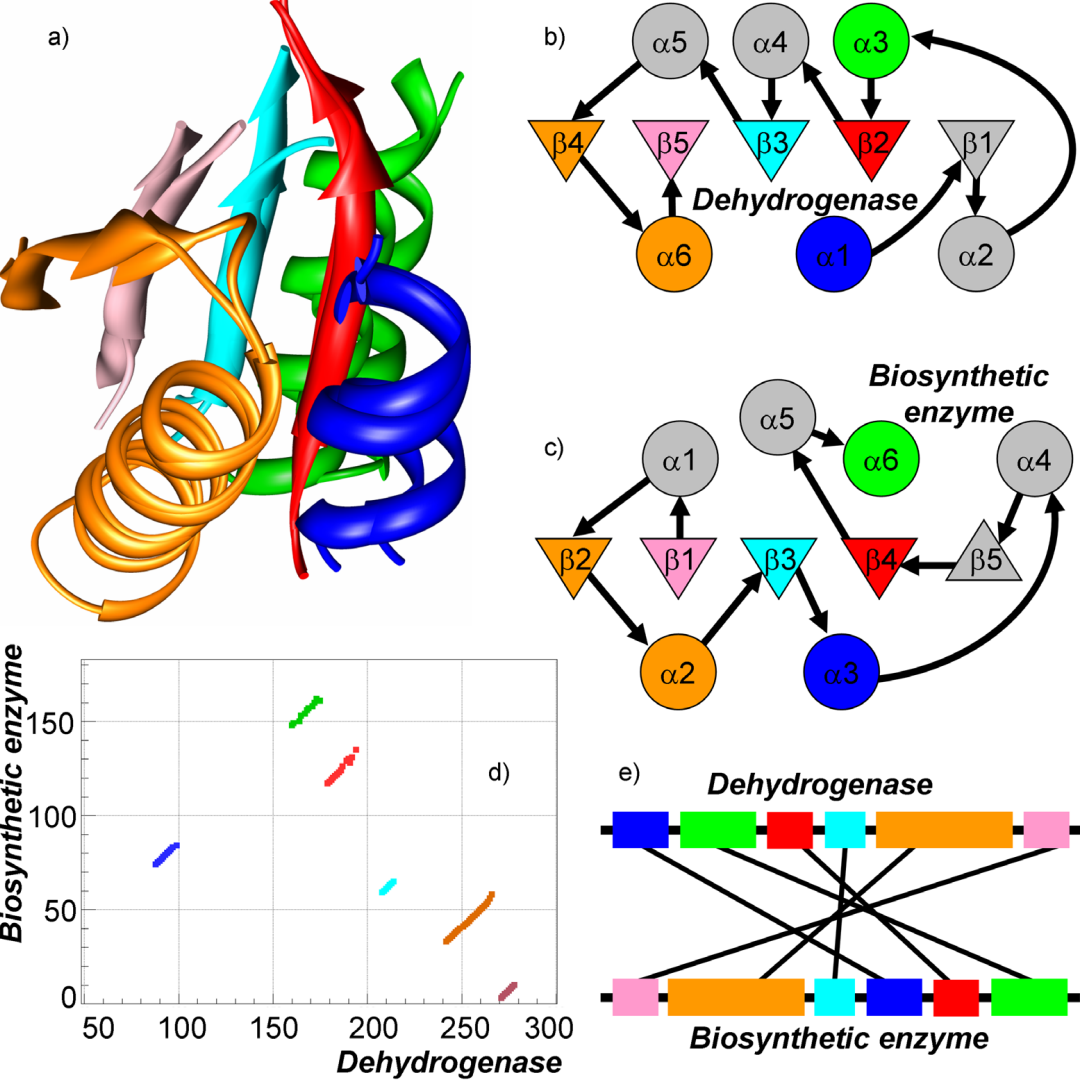
**Example of non-sequential alignment without a dominant sequential part**. Structures of the alpha subunit of 2-oxoisovalerate dehydrogenase (PDB-code 1v16:A) and molybdenum cofactor biosynthetic enzyme (PDB-code 1di6:A) have been aligned by TOPOFIT with *N*_*e*_*/RMSD *of 95/1.6 Å. Both proteins have *α*/*β *structure but belong to different folds: the THDP-fold and to the fold of molybdenum cofactor biosynthetic enzymes respectively. The longest sequential alignment (composed of blue and red segments) has 25 residues. a) displays superposition of the aligned regions. b) and c) display the topologies of the secondary structure elements in the proteins. d) displays the corresponding alignment plot. e) displays a schematic linear diagram of segment permutation in the alignment.

In the above examples there is a common feature: one can clearly identify a long sequential segment in an alignment with the non-sequential part(s) being substantially smaller than the sequential one. While alignments with such a feature occur frequently, nevertheless, we have observed many cases without a dominant sequential part. An example of such case is shown in Figure 7 displaying an alignment of alpha subunit of 2-oxoisovalerate dehydrogenase from *Homo sapiens *(PDB-code 1v16:A) and molybdenum cofactor biosynthetic enzyme from *Escherichia coli *(PDB-code 1di6:A). Both proteins belong to the *α*/*β *class, but to different folds: THDP-fold and molybdenum cofactor biosynthetic enzymes fold respectively. The core of the domains consists of five *β*-strands surrounded by six *α*-helices. In dehydrogenase all strands are parallel while in biosynthetic enzyme one of the strands (namely *β*5) is antiparallel. The structures are aligned with *N*_*e *_= 95 residues and *RMSD *of 1.6 Å. The structural alignment consists of six fragments (Figure 7), one of the fragments contains an *α*-helix and a *β*-strand (22 residues), while the others are single secondary structure elements: *α*-helixes or *β*-strands. Four parallel *β*-strands are well aligned, but their orders in polypeptide chain are completely different (see Figure 7b and 7c), i.e. *β*2 is aligned to *β*4, *β*3 to *β*3, *β*4 to *β*2, and *β*5 to *β*1. The order of *α*-helices is also different in both polypeptides (*α*1 is aligned to *α*3, *α*3 to *α*6, and *α*6 to *α*2). Interestingly, the sizes of the aligned *β*-strands are almost the same, while the sizes of the *α*-helices are different, e.g. helix *α*6 in the dehydrogenase has an extra turn compared to the corresponding helix *α*2 in the biosynthetic enzyme. The longest possible sequential alignment is just 25 residues long, which is less than one third of the entire structural alignment.

**Figure 8:**
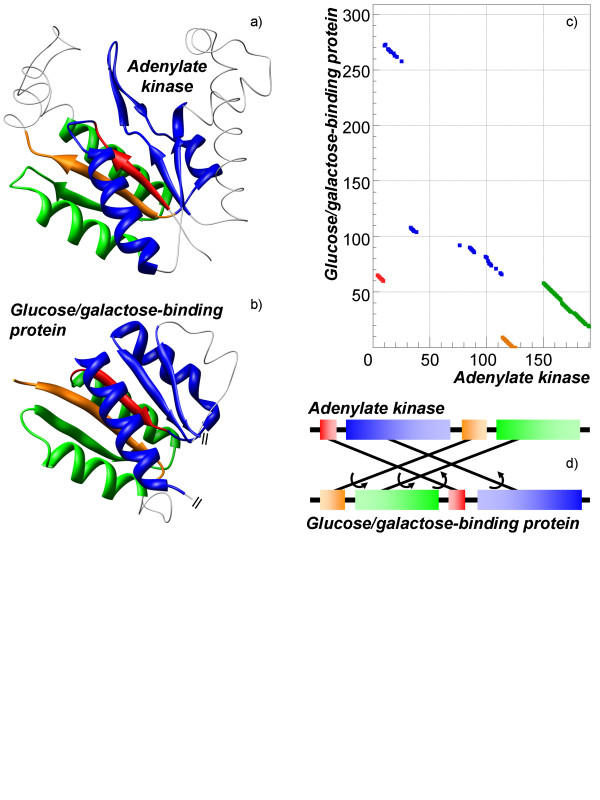
**Example of completely reverse non-sequential alignment**. Structures of adenylate kinase (PDB-code 1ki9:A, shown on a) and glucose/galactose-binding protein (PDB-code 1gca, show on b) have been aligned by TOPOFIT with *N*_*e*_*/RMSD *of 85/1.5 Å. Alignment segments are shown by different colors. In each segment the order of residues is different in the compared protein. c) displays the corresponding alignment plot. d) displays a schematic linear diagram of segment permutation in the alignment.

Another interesting type of alignment is a completely reverse alignment. In this type two proteins share significant structural similarity, while their sequences align in the opposite directions in all the aligned fragments. To the best of our knowledge, only one case of the reverse alignments is well-known; the *α*-helix bundle with several helices, where one or many of the helices can be aligned in the opposite direction. In the presented study many cases of the reverse alignments have been found. A reverse complex alignment of adenylate kinase from *Methanococcus thermolithotrophicus*(PDB-code 1ki9:A) and glucose/galactose-binding protein from *Salmonella typhimurium *(PDB-code 1gca) is shown in Figure 8. The alignment consists of four segments. The longest segment consists of four consecutive fragments: *α*-helix, *β*-strand, *β*-strand, and *α*-helix. In both proteins the segments have long insertions: in the adenylate kinase three helices are inserted between the two aligned *β*-strands, while in the glucose/galactose-binding protein another domain is inserted between the second aligned *β*-strand and last aligned *α*-helix. The fourth segment represents an alignment of consecutive *α*-helix, *β*-strand, and *α*-helix. The remaining two segments represent an alignment of single *β*-strand. This is a remarkable example of how the same structure can be formed by the polypeptide chain going in opposite directions; moreover, the order of the segments forming the structure is different in both sequences.

General statistics on all different alignment types is shown in Table 1 and described in the following sections.

### Alternative alignments

Non-sequential alignments can be trivial if they occur as a result of symmetry or shift in protein structure, but such cases are easily detected: in this case an alternative sequential alignment should exist. It is known that proteins with symmetries and repeats have many alternative alignments, thus, for each protein pair we have evaluated all possible alternative alignments with similar length (Δ*Ne *< 20). Once, an alternative sequential alignment has been found the protein pair was considered to be sequential. Only those non-sequential alignments without any alternative sequential alignments have been considered as true non-sequential cases and are included in the following analysis.

### General classification of non-sequential alignments

We have classified non-sequential alignments between proteins into three classes based on the types of alignment fragments in the alignment: forward (all fragments are of forward type), reverse (all fragment are of reverse type), and mixed (different fragment types). Furthermore, each class has been subdivided into subclasses based on the pattern of fragment permutation: simple (order of fragments is not permuted), circular (cases fitting the definition of circular permutation), swaps (two fragment are swapped but is not a circular permutation), and complex (all other cases). Statistics on the number of non-sequential cases using different thresholds (see **Methods**) and considering alternative alignments have been summarized in Table 1.

As seen from Table  1, the majority of non-sequential alignments (13.2–22.7%) are of the forward class; the number of mixed alignments is smaller but, is still significantly large (3.9–10.7%), while the reverse alignments are much less populated (0.3–1.8%) with only several hundred such cases found. The forward circular alignments is the most populated class, with more than 50% of all non-sequential alignments belonging to this class.

There is a clear tendency that the more complicated alignments are less prevalent for forward and reverse classes, i.e. there are fewer complex than swap alignments, while there are fewer swap than circular alignments. Contrary to this tendency, more complicated alignments in the mixed class are more abundant, i.e. there are more complex than swap alignments, while there are more swaps than circular alignments. Interestingly, the number of simple alignments in this class is of the same order as the number of complex ones, i.e. there is a tendency that if an alignment has two types of fragments (reverse and forward) then it is either very simple (has no permutations) or very complex (has too many permutations) alignment. Table 1 also demonstrates that variation in parameters (using different thresholds and considering alternative alignments) does change the proportion of non-sequential alignments; nevertheless, the proportion remains significant, of the order of 20%. The Table 1 also shows that the usage of different data sets results in comparable numbers, thus, crosschecking the obtained numbers.

### NS alignments occur across many folds, as well as between different folds

Since all structures in SCOP are split into domains and classified, the D2 dataset is better suited for analysis of alignment distribution among protein folds. All alignments can be clearly separated into three groups by dominant type of secondary structure elements of the aligned residues: all-*α*, all-*β*, and mixture of *α*and *β *(see statistics in Table 2). The majority of non-sequential alignments (48%) are found for proteins with a mixture of helices and sheets, while for all-*α*and all-*β *groups the proportion is 24% and 28% respectively. Remarkably, the proportions are not very different from the proportions for all alignments, showing an even distribution of non-sequential alignments in protein classes. Another interesting fact is that consideration of alternative alignments eliminates a large amount of symmetry and/or shift related case (23% of total alignments), with the majority of all-*α*alignments being *α*-helical bundles.

**Table 2:** Distribution of non-sequential alignments by protein classes based on analysis of dataset D2.

Non-sequential	1,130	24%	all-*α*	269	24%
			all-*β*	321	28%
			*α *and *β*	540	48%
Symmetry and/or shift related	1,069	23%	all-*α*	613	57%
			all-*β*	217	20%
			*α *and *β*	239	23%

Sequential	2,449	53%	all-*α*	514	21%
			all-*β*	367	15%
			*α *and *β*	1,568	64%

Total	4,648	100%	all-*α*	1,396	30%
			all-*β*	905	20%
			*α *and *β*	2,347	50%

The line "symmetry and/or shift related" displays statistics for alignments, which are considered to be sequential after analyzing alternative alignments.

The following observations have been made using true non-sequential alignments: 17,428 in dataset D1 and 1,130 in dataset D2 (first row in Table 2). Non-sequentially related proteins have been found in 272 folds and several most frequently found folds with non-sequential alignments are presented in Table 3. While one can see that a lot of non-sequential cases are found for proteins with symmetrical structure, their frequency (of non-sequential alignments) has to be normalized to the occurrence of proteins in a particular fold to allow for proper comparison of numbers. In other words, one has to compare a fraction of non-sequential alignment in each fold. The table shows that a typical fraction of non-sequential alignments within a particular fold, regardless of its symmetry, is of the order of 20–30% (bold columns). Moreover, the fraction of non-sequential alignments for proteins with different folds (30–40%) is of the same order of magnitude as for proteins with the same fold. Interestingly, up to 50% of non-sequential alignments are found for proteins with a different fold, which signifies that non-sequential alignments are not limited to a particular fold or set of folds.

**Table 3:** Distribution of non-sequential (NS) alignments among different protein folds as defined by SCOP.

Fold	% of all NS alignments (dataset D1)	**% of all alignments in fold (dataset D1)**	% of all NS alignments (dataset D2)	**% of all alignments in fold (dataset D2)**
c.1) TIM *α*/*β*-barrel	35.1	**32.5**	25.8	**33.6**
b.69) 7-bladed *β*-propeller	2.8	**66.3**	5.3	**71.4**
c.66) S-ALMD methiltrtansferase	4.4	**34.0**	4.2	**11.2**
b.68) 6-bladed *β*-propeller	1.6	**47.3**	3.4	**70.4**
a.102) *α*/*α *a toroid	0.90	**44.0**	1.8	**46.5**
c.69) *α*/*β*-Hydrolase	0.99	**6.3**	1.4	**4.2**
b.82) Double-stranded *β*-helix	0.50	**15.7**	1.2	**21.9**
b.29) Concanavalin A-like lectins/glucanases	0.74	**34.0**	0.9	**22.7**
b.80) Right-handed *β*-helix	0.2	**11.9**	0.7	**11.8**
d.159) Metallo-dependent phosphatases	0.2	**30.7**	0.6	**35.0**
f.4) Transmembrane *β*-barrels	0.67	**32.1**	0.5	**18.2**
d.142) ATP-grasp	0.01	**7.1**	0.4	**30.1**
a.24) 4-helical up-and-down bundle	1.7	**30.0**	0.4	**17.4**
c.72) Ribokinase-like	0.6	**40.1**	0.4	**40.0**
h.4) Antiparallel coiled-coil	0.52	**15.0**	0.3	**12.0**
c.68) Nucleotide-diphospho-sugar transferases	0.07	**5.7**	0.3	**14.3**
b.67) 5-bladed *β*-propeller	0.2	**48.4**	0.3	**50.0**
c.2) NAD(P)-binding Rossmann-fold domains	6.8	**32.8**	0.3	**21.4**
c.3) FAD/NAD(P)-binding domain	4.4	**22.2**	0.3	**42.9**
Other folds	18.9	**--**	2.5	**--**
Different folds or no fold assignment	18.7	**41.8**	48.9	**31.9**

The line "Other folds" shows the percentage of non-sequential cases in all other folds not in the table. The last line shows the percentages of non-sequential alignments where compared proteins have different fold assignments or do not have an assigned fold.

The table also shows that the numbers, obtained using the two data sets, agree with cases of large discrepancy (e.g. fold of 'FAD/NAD(P)-binding domain') being exceptional. The reason for this is the outdated version of SCOP (dataset D2), when compared to TOPOFIT-DB (dataset D1), and ambiguity in assigning SCOP folds to TOPOFIT-DB's centroids, which are not split into domains and can represent multi-domain proteins. Thus, the discrepancies in numbers are explained purely by technical rather than biological or methodological reasons and results obtained using the two datasets are consistent.

### Protein structure symmetry does not explain non-sequential alignments

**Figure 9:**
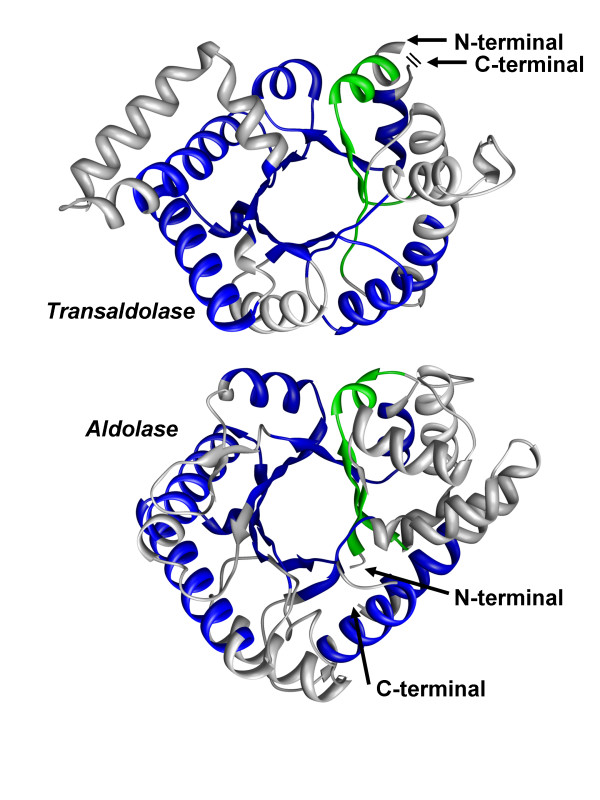
**True biological non-sequential alignment in proteins with symmetrical structures**. Structures of transaldolase B (PDB-code 1onr:A) and class I aldolase (PDB-code 1fba:A) have been aligned by TOPOFIT with Ne/RMSD of 142/1.8 Å. While the structures can be aligned in a sequential way, the best alignment found by TOPOFIT is a circular permutation. The alignment consists of two segments colored blue and green. The segment shown in green is located at C-terminal end in transaldolase, while in aldolase it is located at N-terminal end. The TOPOFIT alignment reflects the correct "biological" alignment as discussed in [43].

While trivial non-sequential alignments (occurring as a result of symmetry or shift in protein structure) had been eliminated, still non-sequential alignments in symmetrical structures have been found. This points to the fact that a non-sequential alignment in a symmetrical structure is not always a trivial case. Consider as an example, the structure alignment of transaldolase B from *Escherichia coli *(PDB-code 1onr:A) and class I aldolase from *Drosophila melanogaster *(PDB-code 1fba:A) shown in Figure 9. Both structures are TIM barrels and can be aligned sequentially preserving the order of *α*/*β*-units (i.e. first *α*/*β*-unit is aligned to first, second to second, etc.) over 170 residues with *RMSD *of 3.6 Å (CE [42] alignment). Most of the alignment methods will agree that such an alignment is statistically significant. However, as discussed [43] the correct "biological" alignment must be a circular permutation, where the first *α*/*β*-unit of transaldolase is aligned to the third unit of aldolase, i.e. there must be a shift by 2 units in the alignment. The best structure alignment for this protein pair produced by TOPOFIT reflects such a circular permutation with 142 aligned residues and RMSD of 1.8 Å. Therefore, this example shows that non-sequential alignment for symmetric protein structures is not necessarily a trivial consequence of symmetry and in fact, can represent the true biological relation between proteins.

**Figure 10:**
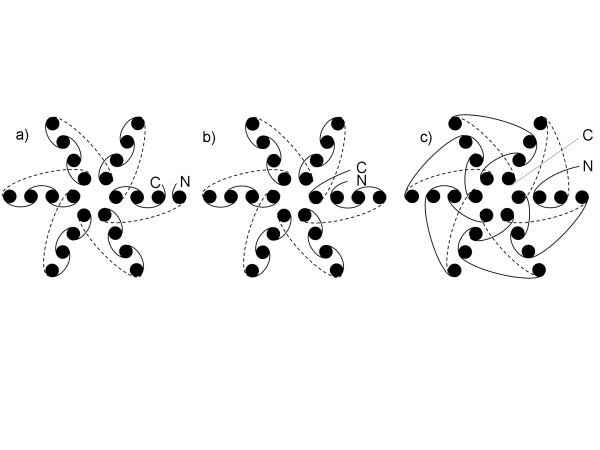
**Different topologies of *β*-strands in the fold of 6-bladed *β*-propeller**. Even though the structures of beta-propeller are symmetrical, none of the proteins with the displayed topologies of *β*-strands can be aligned in a sequential way. Picture is adopted from Figure 1 in [44].

Another interesting case of alignment in proteins with symmetrical structures can be found for proteins of 6- and 7-bladed *β*-propeller folds. Proteins in these folds are characterized by 6 and 7 blade-shaped *β*-strands arranged toroidally around a central axis. Each strand typically has four antiparallel *β*-strands twisted so that the first and fourth strands are almost perpendicular to each other. The majority of non-sequential alignments for proteins of these folds are circular permutations. An important aspect of these alignments is that they cannot be explained by a simple symmetrical shift by a whole number of blades because there is always a non-sequential region inside of a blade consisting of 1, 2 or 3 *β*-strands (see schematic diagram in Figure 10a and 10b). Besides circular permutation, more complex cases of non-sequential alignments can be found while aligning structures of *β*-propeller. The complexity of the alignment arises from different topology, referred to as *β*-pinwheel [44], of *β*-strands in some structures (see Figure 10c). Again, for these cases a symmetrical shift by a whole number of blades does not explain non-sequential alignments. Thus, the unusually high (see Table 3) fraction of non-sequential alignments in *β*-propellers folds is not surprising. Overall, these examples show that indeed one can find true-positive non-sequential alignments in symmetrical structures.

To show that non-sequential cases are found not only in symmetrical structures we have made an additional test. Knowing that 48.9% of non-sequential alignments are found when aligned structures belong to different folds (using dataset D2), we have excluded folds from the analysis where there are at least two proteins with non-sequential alignment. Thus, all potentially symmetrical folds have been excluded resulting in a new dataset (reduced dataset), where all non-sequential alignments occur only between proteins of different folds. It was found that non-sequential cases are found in 7.7% of cases of reduced dataset, which is smaller than 21.2% on the whole data set, but is still very significant. In other words, at least one third of non-sequential alignments are found in non-symmetrical structures.

The previously observed results can be briefly summarized: 1) Non-sequential alignments are found in many non-symmetrical folds; 2) Non-sequential alignments are spread more or less evenly across folds, i.e. there is no specific fold(s) preferable for non-sequential alignments; 3) Up to 50% of non-sequential alignments are found for proteins with different folds; 4) The proportion of non-sequential alignments for proteins with different folds is comparable with proportions for proteins with the same fold; 5) At least one third of non-sequential alignments are found in non-symmetrical structures. Thus, the conclusion is that non-sequential alignments do occur in any class and type of protein structures and a protein structure symmetry/shift does not explain non-sequential alignments. In other words, the occurrence of non-sequential alignments is a general feature of protein structure.

### All possible complexities of fragment rearrangements have been observed

Non-sequential alignments can be very simple that only one fragment is non-sequential, whereas, they can be so complex that only one fragment can be put in sequential order in both sequences. In other words, we have observed very simple and complex rearrangements of structurally equivalent elements in proteins. In order to address rearrangement complexity we introduce the term "rank" of an alignment, which is the number of rearrangements of structurally equivalent parts of proteins needed to put them in sequential order in the sequences of both proteins. According to this definition, sequential alignments are represented as a single structural equivalent and thus have rank zero, while circular permutations and cases similar to the one shown in Figure 1, have rank one and more complex alignments have rank two or higher. Technically, we have calculated rank as the number of segment rearrangements rather than fragment rearrangements (see **Methods**). This was done to ensure that rank is not overestimated due to the presence of several fragments in one segment. Using this definition, it is easy to see that any alignment with *n *fragments can have the highest rank of *n - 1*, because at least one structural element is not rearranged relative to others (we do not consider reverse alignments here).

**Figure 11:**
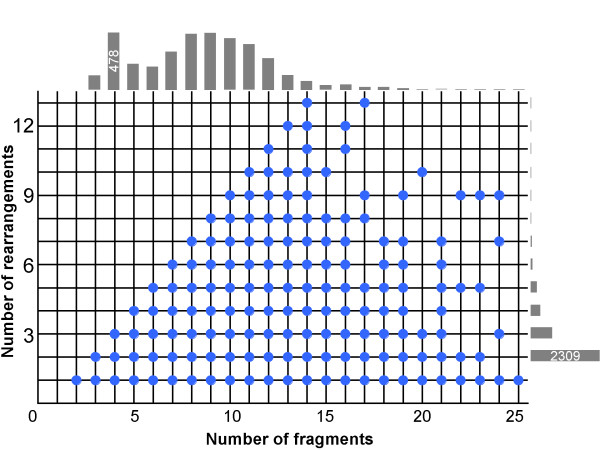
**All possible complexities of fragment rearrangements in an alignment have been observed**. The figure shows a scatter plot of the number of fragment rearrangements vs the number of fragments in the alignment. Bar charts on the top and on the right of the picture reflect the occurrence of alignments with a particular number of fragments and number of rearrangements. Only alignments with more that one fragment rearrangement have been considered to calculate the bar proportion. The numbers on the bars help visualize the scale. The area in the right-upper corner is not populated because of a lack of statistics (see text).

Figure 11 shows a scatter plot of alignment rank vs number of fragments. As seen from Figure 11, for alignments consisting of up to 14 fragments almost any complexities, i.e. any possible rank value (with rare exceptions) has been observed. For alignments with a larger number of fragments this is not the case, but it can be explained by the limited statistics (see bar charts on the top and the left of the picture). Thus, we hypothesize that there is no restriction on how elements of protein structure can be permuted in a sequence and that any rearrangement of fragments can be found in nature. An illustrative example of an alignment with many rearrangements has already been described in Figure 7.

### Analysis of the redundant data set

It is interesting to understand whether there are any non-sequential cases in highly similar proteins, both in structure and in sequence, i.e. those that have been grouped in TOPOFIT-DB in clusters. Thus, alignments between the structures of each of 8,865 clusters have been collected for a total of 2,509,599 alignments. The analysis reveals that the absolute majority of detected non-sequential cases are circular permutations with few exceptions. Statistically, 31,358 out of 2,509,599 alignments were non-sequential, out of which 95.5% (29,938 cases) were circular permutations, 3.5% represented alignment of different conformation of same protein, and the remaining 1% have been accounted for non-sequential alignments in only 7 protein families: fructose-1,6-bisphosphatase (1fpk:A and 1d9q:B), arrestin (1cf1:A and 1ayr:B), annexin (1hm6:A and 1hvg), aspartate/ornithine carbamoyltransferase (2atc:B and 1rac:B), 3-isopropylmalate dehydrogenase (1iso and 1hqs:A), NADH peroxidase (1f3p:A and 1nhs), *α*-*β *tubulin (1jff:B and 1tub:B). Thus, we can state the absolute majority of proteins with high sequence similarity have only circular permutations cases of non-sequential alignments.

## Discussion

In the presented study a comprehensive large-scale analysis of non-sequential alignments between all PDB structures (as of July 2005) has been performed. We have found that up to 35.2% of all significant alignments are non-sequential. Consideration of different thresholds and alternative alignments has been made to ensure robust detection of non-sequential cases. These variations in methodology revealed that non-sequential alignments are found in at least 17.4% of cases. Thus, the estimated proportion of non-sequential alignments is in the range of values between 17.4 to 35.2%, which is a significant proportion of structural relations not detected by most of the current methods.

It was found that the majority (more than 50%) of the non-sequential alignments fit to the formal definition of circular permutation. It is important to stress here how this number should be understood. Often, proteins aligned in a circular way are assumed to be evolutionary related and this assumption is often encoded into an alignment method to detect such cases. There is no such assumption (of evolutionary origin) in the methodology used in this study and thus, a large number of circular alignments alone does not necessarily mean an evolutionary relationship between the compared proteins. The same way, the origin of more complex non-sequential alignments is not clear.

Besides circular permutations, non-sequential alignments with a large variety of alignment patterns have been found. All possible complexities of rearrangements, various sizes and numbers of non-sequential fragments have been observed. It has been found that non-sequential alignments are not limited to proteins of any particular fold and are present in more than two hundred of different folds. Moreover, up to 50% of non-sequential alignments are found for proteins with a different fold assignment. While many of the non-sequential alignments were found for proteins with symmetrical structures, it has been shown that protein structure symmetry does not explain non-sequential alignments. Therefore, compelling evidence of different forms has been provided, confirming that non-sequential alignments between proteins are diverse and widespread across the protein universe.

Many cases of reverse alignments in various folds have been found in this study. To the best of our knowledge, only one case of reverse alignment is well known, the *α*-helix bundle with several helices, where one or many of the helices can be aligned in the opposite direction. The *α*-helix bundles have been studied experimentally and successful attempts on redesigning the four-helix bundle to have inverted helices have been reported [45,46]. Such successful redesign of *α*-helix bundle can be theoretically extended to other protein folds with the cases of reverse alignments observed in this study. Thus, the existence of the reverse alignments for proteins of other folds can serve as the basis for new approaches in protein engineering to redesign proteins.

The discovery of the existence of all theoretically possible complexities of fragment rearrangement in proteins is intriguing (see **Results **and Figure 11). The plot is not complete due to limited statistics, which we assume as of the lack of the data for the large proteins. We believe that there is a strong confidence in a statement that any possible combination of fragments can be found in any protein structure. Currently, one can introduce a hypothesis to test (with strong support from all the presented results), which can be formulated as follows: the three-dimensional shape of tertiary structure does not depend on the order of protein fragments in the polypeptide chain, the protein core has just to be organized in a complementary manner and internal fragments have to fit to each other, while the external loops might reconnect the internal fragments in any reasonable way. The protein core here is the structural invariant, which was introduced earlier in our TOPOFIT method [39], while the external loops are the fragments outside of the structural invariant.

Such a hypothesis can be tested experimentally and will provide a strong empirical basis for protein redesign as a recombination of different fragments; one can see many practical applications from it to create new proteins. The validation of the hypothesis will broaden our understanding of protein structure organization and folding, and can be directly applied in fragment-based methods for protein structure and function prediction [47]. It is encouraging that the hypothesis is supported by experimental studies on circularly permuting protein structure [20-30] and redesigning four-helix bundle proteins to have several different topologies of helices [45,46]. Therefore, a similar reengineering by rearranging fragments may be applied to other protein folds.

## Conclusion

The discovery of the widespread occurrence of the non-sequential alignments among many different protein folds presents an interesting phenomenon. Based on this phenomenon, one may suggests that there is some unknown common rule that governs relations between proteins detected by the non-sequential alignments, a missing rule(s) in our understanding of protein structure organization. Finding such a rule can be a challenge for the future research, but, apparently, the existence of the non-sequential alignments is not rare effect but rather a systematic feature of all proteins. More detailed studies of these alignments will bring new insight in our understanding of protein evolution, protein stability and protein folding and functionality. As a first step toward understanding the non-sequential alignments, a testable hypothesis has been suggested, stating that the three-dimensional shape of protein structure does not depend on the order of protein fragments in the polypeptide chain.

## Methods

### Selecting representative data sets

For this study the structural relations between the representative proteins from the TOPOFIT-DB [40] database (centroids), have been analyzed. The data set from TOPOFIT-DB contains all 33,315 proteins from PDB (as of July 12, 2005). All structures in the database are divided into clusters of high similarity, both in structure and in size, with assigned (to each cluster) centroids representing each cluster. The 8,865 protein clusters in TOPOFIT-DB can be considered as an analog of a structural families in CATH [48] and SCOP [41]. For each cluster a centroid structure is chosen as a representative by maximum sum of *Z-scores *to all other proteins in the cluster. Comparison of the centroids and proteins inside each cluster resulted in 39,276,862 structural alignments stored in the database. For this study, only centroid-centroid alignments from TOPOFIT-DB with *Z-score *> 7 have been used, leading to a total of 82,263 alignments.

A second data set has been collected by comparing alignments between protein families as defined by SCOP (release 1.69). For each family the first structure, in the list of proteins assigned to the family, has been used as a representative, resulting in 2,845 representatives. 4,045,590 structural alignments have been produce and stored in TOPOFIT_DB database [40] by comparing the representatives. For this study, only alignments with *Z-score > 7 *have been used, leading to a total of 4,648 alignments.

### Identifying sequential parts (segments) and noise filtering procedure

Since TOPOFIT alignments can be fragmented we define alignment fragment as the sequential part of an alignment without "long gaps", gaps longer than 2 residues. The cut off has been chosen based on the analysis of gap distribution in all alignments. Then we define an alignment segment as a sequential (reverse or forward) part of a structural alignment (see Figure 1). An alignment segment is different from an alignment fragment as the segment can have long gaps (longer than 2 residues) and consequently, may consists of one or more fragments. Thus, a fragment is a particular case of a segment. In Figure 1 segments are highlighted in different colors. For simplicity only the term "segment" is used in the following description of the procedure. During the procedure some alignment residue pairs were considered as noise and removed (circled on the figure). Let us define an interfering segment ***z***, for a pair of segments ***x ***and ***y***, as a segment located in between the two segments in either of the sequences (see example on the Figure 1). The input parameter in the algorithm is the value of *F*_*min*_, which controls the minimal size of a segment, i.e. all segments smaller than *F*_*min *_are eventually removed from the alignment or combined with other segments.

Alignment segments have been combined in a pairwise manner as follows. On each step all pairs of segments have been evaluated by the following three values (by criteria pointed in parenthesis):

1) number of segments interfering with it (smaller preference);

2) number of aligned residues in the interfering segments (smaller preference);

3) cumulative number of residues in the tested pair of segments (larger preference).

The best pair is found by comparing those values, where each next value is used only if the preceding values were equal. Segments in the best pair are combined only if the pair has no interfering segments. Otherwise, the interfering segment having a minimal number of aligned residues is removed from the structural alignment. So, on each step, the number of segments decreases by one. Steps are repeated until all segments are combined into one or the segment to remove has length more or equal then value of *F*_*min*_.

The procedure considers forward and reverse segments simultaneously, however only segments of the same type (both are either forward or reverse) are being combined. Special care is taken with segments of length one; they are evaluated in pairs with both forward and reverse segments. Here it is important to stress that the minimal fragment parameter *F*_*min *_is not like a conventional threshold because short fragments are not simply removed from the alignment, but first are tested for the possibility of being combined with longer fragments and only upon failure are removed.

### Robustness of non-sequential alignment detection, signal/noise discrimination, optimal values of *F*_*min*_

**Figure 12:**
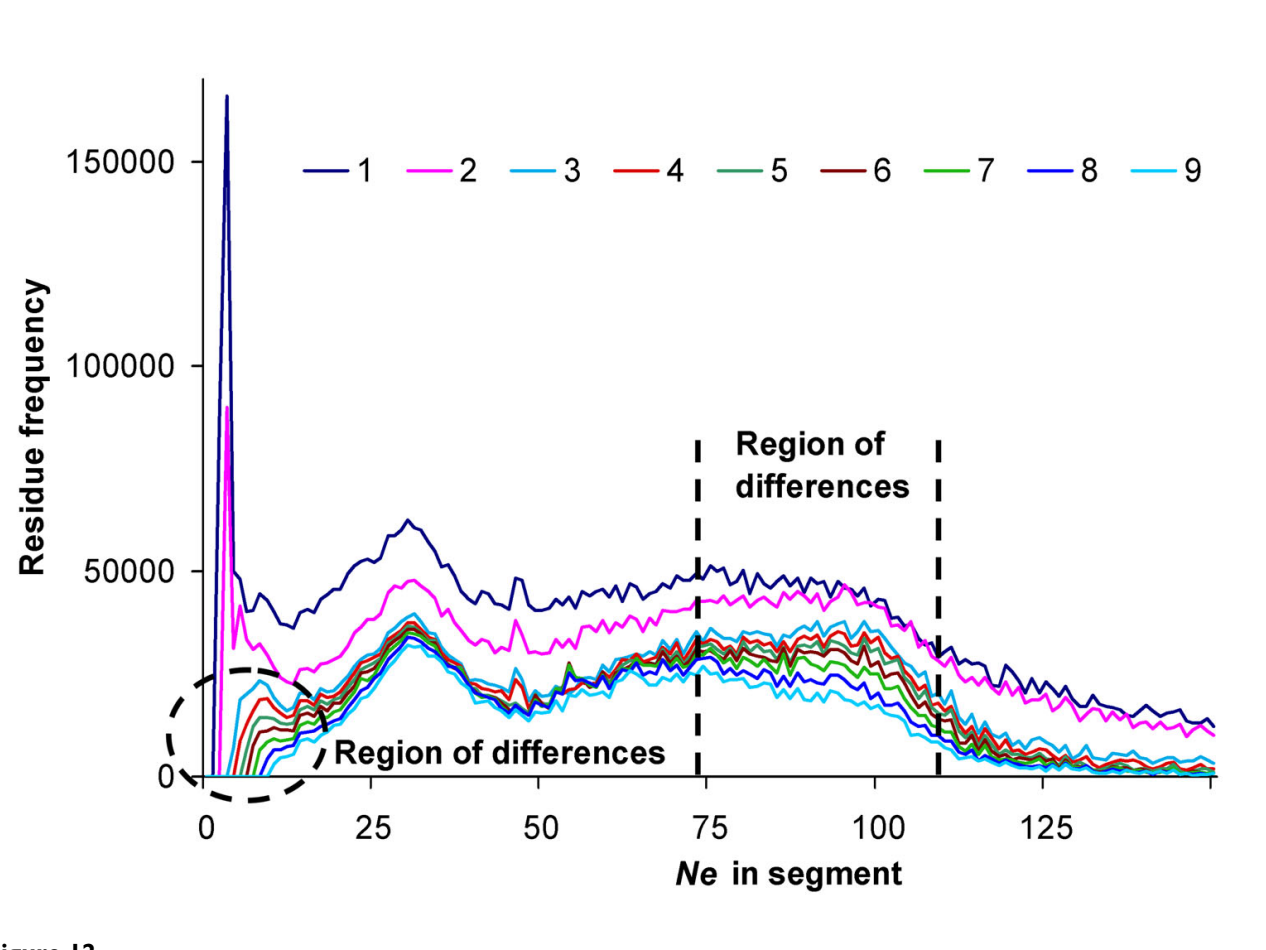
**Distribution of the number of residues in segments with particular *N*_*e*_**. Different colored curves show distributions obtained for different minimal length (*F*_*min*_) of resulting segments. The most dramatic changes occur when the value changes from 2 to 3 residues, clearly identifying the threshold for noise discrimination. The distributions are almost the same for values 3–6 of *F*_*min*_, while for higher values, the distributions start to deviate from each other (marked as regions of differences), thus identifying the threshold for clear signal separation. Therefore, the optimal value of *F*_*min *_for analysis should be between 3 and 6 because here is where the noise is eliminated without significant affect on the signal.

The TOPOFIT method has no limitations on fragment size and some fragments can be as small as a single pair of aligned residues, which is illustrated as single dots in the alignment. Such aligned pairs of residues can be signal or noise (see Figure 1). Therefore, while finding and analyzing alignments care must be taken to discriminate between the two. Signal to noise discrimination has been achieved by applying the procedure of combining alignment fragments into continuous alignment segments (described above). The frequency distributions of residues in the segments for the range of *F*_*min *_values have been calculated in order to evaluate the discrimination of noise caused by small size fragments (see Figure 12). The blue line shows the original distribution when the value of *F*_*min *_= *1*. Distributions with gradually increasing minimal fragment have also been produced for values of *F*_*min *_equal to 2, 3, 4, 5, 6, 7, 8 and 9 residues.

The major change in distribution occurs at *F*_*min *_changing from 2 to 3. Not only has the area under the distribution changed dramatically (i.e. number of non-sequential cases reduced), but the spike in the distribution at lower values has disappeared. Thus, it is evident that the noise is mostly represented by short fragments of length 1 and 2 residues. The distributions for *F*_*min *_values from 3 to 6 do not differ much, while larger *F*_*min *_values lead to significant disruptions in the shape of distributions in the region from 75 to 110. Consequently, non-sequential alignments mostly consist of aligned segments of 6 or more aligned residues. Therefore, the best signal-to-noise discrimination can be archived when the value of the *F*_*min *_parameter equals 3–6 residues. This is where the majority of the noise is filtered out while the signal (quantity of non-sequential alignments) is not cut. In the overall analysis presented here, the value *F*_*min *_= 4 has been used, while additionally a tightened criteria, *F*_*min *_= 6, has been applied for cross checking.

Applying tightened criteria resulted in an 11 % decrease (25,849 compare to 28,949) in the number of non-sequential cases detected. Thus, we concluded that at selected values of the *F*_*min *_parameter, detection of non-sequential cases is robust.

### Alignment rank

The rank of an alignment is defined as the number of rearrangements of structurally equivalent parts of proteins needed to put them in sequential order in the sequences of both proteins. Technically, the rank was calculated as the number of segment permutations. In order to calculate the number of permutations in an alignment, the corresponding alignment segments have been ordered by sequence order in the first aligned protein and numbered incrementally starting from one. Then, the segments have been ordered by sequence order in the second aligned protein. In case the considered alignment is non-sequential, renumbering will permute the order of the numbers assigned. For example, the order of numbers for the alignment shown in Figure 1 will be *(1,3,2,4)*. A simple bubble sort algorithm has been used to calculate the number of permutations needed to sort the numbers in ascending order. For the alignment shown in Figure 1 only one permutation is needed. For reverse alignments, a reverse order of amino acids for second sequence has been considered while calculating permutations and for mixed alignments, a reverse order of amino acids for the second sequence has been considered only if the cumulative *N*_*e *_of reverse segments is higher than the cumulative *N*_*e *_of forward segments.

### Data analysis

The non-sequential alignments were visualized and analyzed in integrated software package, Friend [49] with the integrated TOPOFIT method [39]. The final views (shown in figures) of proteins structures were produced with Chimera [50]. Data analysis has been performed with the aid of the ROOT software package [51]. All data are publicly available in TOPOFIT-DB and can be accessed at our web site [40].

## Authors' contributions

AA did the data collection, calculations, and analysis and prepared the manuscript. VAI did design of the project, data analysis and prepared the manuscript. All authors have read and approved the final manuscript.
